# The introduction of workplace-based assessment into postgraduate medical training in South Africa: trainee perspectives

**DOI:** 10.1186/s12909-026-08792-w

**Published:** 2026-02-21

**Authors:** Emma Daitz, Louis S Jenkins, Jacques Janse van Rensburg, Madeleine Muller, Veena S Singaram, Richard Cooke, Sumaiya Adam, Dini Mawela, Gerda Botha, Thakadu Mamashela, Tashneem Harris, Eric Buch, Lionel Green-Thompson, Vanessa Burch, Tasleem Ras

**Affiliations:** 1 Department of Family, Community and Emergency Care, Faculty of Health Sciences, University of Cape Town, Cape Town, South Africa; 2 Department of Family Medicine, Faculty of Health Sciences, Stellenbosch University, Stellenbosch, South Africa; 3 Department of Diagnostic Radiology, Faculty of Health Sciences, University of the Free State, Bloemfontein, South Africa; 4 Department of Family Medicine, Faculty of Health Sciences, Walter Sisulu University, East London, South Africa; 5 Health Professions Education Unit, College of Health Sciences, University of Kwazulu-Natal, Durban, South Africa, eThekwini, South Africa; 6 Department of Family Medicine and Primary Care, Faculty of Health Sciences, University of the Witwatersrand, Johannesburg, South Africa; 7 Department of Obstetrics and Gynecology, Faculty of Health Sciences, University of Pretoria, Tshwane, South Africa; 8 Department of Paediatrics and Child Health, Faculty of Health Sciences, Sefako Makgatho Health Sciences University, Tshwane, South Africa; 9 Practice of Medicine, Faculty of Health Sciences, Sefako Makgatho Health Sciences University, Tshwane, South Africa; 10 Department of Forensic Pathology, Faculty of Health Sciences, University of Limpopo, Polokwane, South Africa; 11 Department of Otolaryngology, Faculty of Health Sciences, University of Cape Town, Cape Town, South Africa; 12 Colleges of Medicine of South Africa, Cape Town, South Africa; 13 Committee of Medical Deans and Deanery, Pretoria, South Africa; 14 Faculty of Health Sciences, University of Cape Town, Cape Town, South Africa

**Keywords:** Specialist medical education, Workplace-based assessment, Resource-constrained setting, Learning environment, Feedback

## Abstract

**Background:**

South Africa (SA) is moving towards implementing workplace-based assessments (WBA) in all medical specialist training programmes in the country. There are many challenges with implementing WBA, with existing literature suggesting implications for resources, and recognizing regulatory, educational, and social complexities. Research on WBA practices, experiences, and perceptions in the SA healthcare system is limited. The aim of this study was to identify factors that could impact WBA implementation from the perspectives of medical doctors in SA undergoing medical specialist training. The findings report on the perspectives, aspirations, and concerns of these postgraduate medical specialist trainees (also known as registrars and residents).

**Methods:**

This paper reports on the qualitative data generated from a longitudinal mixed methods study that employed focus group discussions (FGDs) to gather data at different points in time of WBA implementation. We conducted two phases of institution-specific, interdisciplinary FGDs at seven universities in two phases. FGDs typically included between 6 and 10 trainees, lasted 60 min, and were facilitated online by a sociologist (ED). Sessions were audio-recorded and transcribed verbatim. Data were analysed thematically and inductively.

**Results:**

Six themes were identified from the data. Trainees had a generally positive attitude towards WBA in theory. However, they expressed anxieties about supervisor bias, unequal clinical contexts, and lack of standardization affecting their assessment outcomes if WBA was to be fully implemented. They reported that consultants were often unavailable for WBA activities and misunderstood the differences between summative and formative assessments.

**Conclusions:**

Trainees support WBA in principle but anticipate uneven implementation without structured faculty development, protected observation time, and safeguards for fairness across settings. Early implementation should prioritize role clarity, feedback skills, and context-sensitive quality assurance.

**Supplementary Information:**

The online version contains supplementary material available at 10.1186/s12909-026-08792-w.

## Background

Over the last fifty years, postgraduate medical specialist training has undergone a major shift from a traditional approach which focused on ‘lists of knowledge/skills objectives’ to an outcomes-based approach which centres on graduate competence [1]. Today, competency-based medical education (CBME) is the pre-eminent approach to education in many parts of the world where medical specialists are trained. WBA is part of CBME which takes place as part of day-to-day clinical work [2]. South African medical specialist training institutions are in the process of implementing WBA as a core component of their assessment framework. Contextual and supervisor perspectives have been reported [3, 4]while the perspectives of trainees, those at the receiving end of these innovations, have not yet been explored.

CBME is organized around the context-sensitive functions needed to practice medicine, and the dual convictions that all medical trainees can become competent in the basic performance objectives which can, in turn, be empirically assessed [5]. Over the past two decades there has been a growing interest in, and implementation of, CBME among various stakeholders including educators, clinicians, and policy makers. This is driven by concerns about physician preparedness, patient safety, and public accountability as healthcare systems evolve and become more complex [6, 7]. 

WBA is the mechanism developed to assess trainee competence within the conceptual framework of programmatic assessment, gathering real-time data of clinical behaviour and performance in the workplace [2]. The goal is to inform the supervisor and trainee regarding trainee strengths and areas needing further development so that training can be tailored to specific trainee needs. The information gathered this way – through direct or indirect observations within the workplace – can then be used either formatively to guide trainees’ development, or to make legitimate high-stakes summative decisions at the end of a training period [2]. 

Feedback is the cornerstone of the formative dimension of WBA [8, 9]. Early evidence from the SA context shows that WBA enhances teaching and learning and improves the ability of the supervisor and trainee to engage productively with each other in the workplace [8]. In their study of the impact of WBA across surgical specialties at the University of KwaZulu-Natal, Baboolal and Singaram [8] noted that, WBA and feedback were ‘associated with high ratings for the quality of trainee supervision.’ International evidence also supports the central role of high-quality feedback in WBA [10, 11]. 

Creating a supportive feedback culture within the clinical environment is thus crucial for the success of WBA, but this is also an area of significant complexity in SA. As early as 2011, Bezuidenhout and colleagues [12] reported that relationships between supervisors and trainees may not always be functional or healthy, a finding mirrored in the international literature [13]. More recently, Bagwandeen and Singaram [14] have shown that demographic factors such as gender have a negative impact on the provision of feedback in postgraduate medical education. Thackwell et al.’s [15] research showed that black trainees particularly experience daily and institutional forms of racism during their training. Khine and Hartman’s [16] study showed that while students are creative in dealing with the difficulties they face during training, they experience ‘race, language, departmental culture and social identity as barriers in their learning’ as well as the ‘lack of structured formative training with feedback, evaluation, personal mentoring, and supervision’. Supervisors confirm that bias, favouritism, and a lack of high-quality feedback are potential problems in the training and assessment of medical specialists [3]. 

Issues concerning trust/power dynamics in the feedback component of WBA are not limited to the SA context and have been reported on extensively in the international literature. Research from the international context indicates that real or perceived power imbalances between supervisors and trainees influence students’ willingness to engage with assessment and feedback; [13] that the quality of the supervisor-trainee relationship significantly shapes the success of the implementation of WBA; [17] and that poor relationships can hinder honest feedback and reflective practice [18]. 

Globally, debates about WBA and programmatic assessment in health professions education converge on several recurrent themes. WBA has been championed as a way to integrate assessment *for* learning with assessment *of* learning [19], but its implementation raises concerns about feasibility (workload, documentation burden, and institutional readiness) [20], rater bias [21], and the quality of feedback and decision-making [22, 23]. WBA advocates argue that meaningful triangulation of diverse assessment data can create more valid and reliable judgements of competence than isolated tests alone [24]. However, critics note tensions between formative and summative purposes, information overload, and the need for a shared understanding of the philosophy and processes across stakeholders, including trainees, faculty, and committees [25]. Trust and power dynamics feature strongly: effective systems depend on trusted supervisory relationships and credible competence committees, yet hierarchies and cultural norms can shape feedback cultures and influence assessor behaviour and trainee engagement, potentially introducing bias or defensive practices [13, 26]. 

South African studies contribute substantively to these international debates by contextualizing them within a resource-constrained, culturally diverse, and inequitable health system setting. Recent national surveys and qualitative work among South African medical specialist educators identify high levels of conceptual knowledge about WBA but variable implementation practices and significant structural barriers such as clinical workload, inadequate time and infrastructure, and limited staff capacity for standardized assessment and feedback [3, 4]. Researchers highlight interpersonal dynamics—bias, favouritism, and the quality of supervisor-trainee relationships—as central to successful implementation, echoing global concerns about trust and power but framed within local professional cultures [14, 27]. There is also emerging evidence from SA that WBA usage correlates with improved perceptions of supervision quality and feedback effectiveness, even in constrained settings [27], suggesting that well-integrated WBA may support learning and programme quality where formal assessments have been historically weak. Moreover, research on South African learning portfolios and Entrustable Professional Activities (EPAs) development demonstrates how programmatic approaches can be adapted to decentralized training contexts, informing global understanding of how to balance feasibility, cultural sensitivity in feedback, and quality assurance mechanisms in LMICs [4, 14, 28]. 

The examining body for medical specialist certification examinations in SA, the Colleges of Medicine of South Africa (CMSA), and the South African Committee of Medical Deans (SACOMD), which represents all health science faculties in the country, are currently jointly driving a process of implementing WBA as a key component of the assessment framework in the training and licensing of medical specialists. Recent research has established a baseline for how supervisors perceive the issues alluded to above, along with a range of other issues pertinent to WBA [3, 4]. The question of how WBA is perceived and experienced by trainees remained unanswered. This study aimed to hear their voices by exploring their perspectives and experiences of WBA as it has been implemented to date.

## Methods

This study represents phase 2 of another prospective, multi-centre, mixed-methods longitudinal study which examined factors that could impact WBA implementation from the perspectives of medical specialist educators. This study used a longitudinal qualitative methodology, employing focus groups at two distinct points in time to describe trainee perspectives at varying stages of WBA implementation. 

### Setting

The postgraduate medical specialist training context in South Africa spans 9 universities, with a 10th expected to start training soon. Training typically spans 4–5 years and is guided by a national discipline-specific curriculum. These curricula have typically been developed by consensus-driven processes under the mandate of the training universities and the CMSA, the legislated examining authority for specialists in South Africa. The CMSA oversees the final summative assessment for all medical specialists, a requirement for licensing. The national assessment occurs in co-ordination with universities, who carry the mandate for training. Universities co-ordinate via an umbrella body, Universities South Africa (https://usaf.ac.za/), while the health sciences faculties co-ordinate via a subcommittee of this organization, the SA Committee of Medical Deans (SACOMD). The universities have formal partnerships with the regional health authorities who run state-funded clinical platforms (hospitals, laboratories, clinics, and community-based organizations). Postgraduate trainees, qualified medical doctors who have completed two years of internship training and one year of obligatory community medical service, are appointed as postgraduate students at these universities where they are usually employed by the local health authority or National Health Laboratory Service in the case of trainee pathologists to work on the clinical platform for the duration of their training period. It is in the context of the clinical training platform that universities are implementing WBA, which will feed into the assessment framework of the CMSA.

### Population and sampling

The study population consisted of postgraduate trainees from training programmes offered in twenty-nine specialties at seven institutions in South Africa. The study sites were purposively selected, based on a decision by individual programme directors who volunteered to serve as research pilot sites. Thirty-two unique pilot sites were identified, and their baseline assessments and supervisor perspectives have been reported elsewhere [3, 4]. Separate whole-day, in-person onboarding workshops were held for faculty and trainees at each pilot site. This study focuses on trainee perspectives, with faculty perspectives captured separately elsewhere.

All trainees who attended the onboarding workshop were invited to participate in two focus groups: one approximately one month after the introductory workshop, and second one approximately eight months later. The focus groups were specific to an institution, not a discipline, and were therefore inter-disciplinary but not inter-institutional. The data generation occurred between February 2024 and December 2024.

The desired sample size was based on a combination of representation from all pilot sites, as well as data saturation emerging from the focus group discussions.

#### Inclusion criteria:

1. Registered as a postgraduate student at one of the participating institutions.
2. Trainee should have attended the trainee onboarding workshop.

#### Exclusion criteria:

3. Not available during the data collection period.
4. Trainee not part of one of the selected study sites.
5. Trainees did not participate in the onboarding workshop or other parts of the study.

### Data collection

Data was generated using online focus group discussions (FGD) in English (see addenda A and B), facilitated by ED, an experienced qualitative researcher not involved in any of the training programmes. The FGD were held on zoom. ED is not a medical doctor and has a background in Sociology. An interview guide was developed that aligned with the study objectives and was reviewed by the entire research team. After comments, the guide was implemented with the first FGD and reviewed by ED and TR the principal investigator. No changes were made, and this first FGD was included in the dataset. FGDs were held per institution within one month (phase 1) after the introductory workshop, and a second round approximately eight months after this first FGD (phase 2). Eleven (*n* = 84) FGDs were held in phase one and seven (*n* = 45) in phase two, though the numbers of participants differed. The FGDs were audio-recorded and transcribed by a professional transcribing service. Thereafter, they were checked for accuracy by ED and anonymized before being included in the dataset.

### Data analysis

Thematic analysis was applied to the dataset using Braun and Clark’s six-step process [29] following an inductive approach. During the onboarding workshop and subsequent FGDs, ED made field-notes documenting her observations, especially of contextual and non-verbal occurrences. ED then immersed herself in the audio recordings of each FGD, to ensure that there was familiarity with each recording. The transcripts were then read iteratively by ED and TR, and the initial transcripts were coded independently. This was followed by a discussion between ED and TR on their coding, and consensus was reached on areas of divergence. ED then commenced coding the entire dataset.

Subsequent transcripts were then coded by ED, with emerging codes and themes iteratively discussed within the research team to ensure conceptual coherence and analytic depth. Rather than seeking mechanical inter-rater reliability across the full dataset, consensus was achieved through ongoing reflexive dialogue, comparison of interpretations, and alignment of themes with the evolving dataset. This approach is consistent with reflexive thematic analysis, where rigor is demonstrated through transparency, analytic reflexivity, and theoretical saturation rather than repeated double-coding, and is particularly appropriate for large, longitudinal datasets in complex educational contexts [28]. The codes were then categorized based on their similarity to each other. Once categorized, ED and TR reviewed all the categories and generated themes where the codes within a category were deemed to be cohesive. Important codes that did not fit into a theme were reported independently.

To ensure data quality, we applied the trustworthiness criteria described by Lincoln and Guba [30]: credibility, transferability, dependability and confirmability. We ensured credibility by consistently linking our findings to the actual raw data and presenting quotations to substantiate our findings. Our field notes assisted in triangulating this by providing context for the specific statements of participants. We provide a thick description of the context in which this study took place to approach transferability. Dependability was enhanced by the iterative nature of data checking, review of analysis, and ensuring that audit trails are available for all aspects of the study, including the findings. We engaged with confirmability by including deep reflexivity for the entire study process, reflecting challenges within the data gathering process with the larger group of collaborators, as well as on our own positionality as researchers and practitioners in the field of medical specialist training.

### Reflexivity statement

As part of the study conceptualization and design, we recognized that the authors were all in positions of relative power within the community of medical specialist trainers in SA. This was addressed by appointing ED, a sociologist with no medical training, to lead the process of collecting and analysing data. The intention was to ensure that participants did not feel overwhelmed or intimidated by the realities of the hierarchy that exists within the medical community. Additionally, the workshops and FGDs for supervisors and trainees were held separately, to allow each group to freely express themselves without fear of intimidation or retribution. When the final analysed dataset was presented to the author-group, there were no identifying characteristics included, to ensure that individual institutions or programmes were not identifiable.

### Ethical considerations

The study conforms to the principles of the Declaration of Helsinki (2024). Research methodological review was performed by departmental reviewers, while ethical review and approval took place independently via the institutional review boards of the participating universities. All participants signed an informed consent form prior to being included in the FGDs. All data was completely anonymized of participant, discipline and institutional characteristics, and is stored on a password-protected computer for a total of five years from date of publication of this study.

## Results

Nine universities were included in the study. Due to internal operational challenges two universities could not generate or submit data and were therefore not included in the data set. A total of 11 FGD with 84 participants were held in the first phase of data generation, and 7 FGD with 45 participants in the second phase. They were enrolled across the disciplines of family medicine, obstetrics and gynaecology, forensic pathology, internal medicine, anaesthesiology, paediatrics, otorhinolaryngology, neurosurgery, general surgery, haematological pathology, ophthalmology, clinical pathology, plastic surgery, virology, emergency medicine, and chemical pathology.

We found that data saturation was achieved early in the data generation process. By the fifth interview, meaning saturation was reached, defined as the point at which no new insights, dimensions, or interpretive understandings of existing themes emerged. Subsequent interviews confirmed and enriched existing interpretations without adding novel conceptual insights. This indicated that sufficient depth and richness had been achieved to support robust analysis and interpretation of the study findings. Saturation was assessed through an iterative and reflexive analytic process conducted alongside data collection. After each interview, transcripts were reviewed and coded, with analytic memos used to document emerging interpretations, refinements to existing themes, and points of conceptual uncertainty. Data collection continued thereafter to ensure adequate and planned representation across institutions.

### Emergent themes

**Fig. 1:**
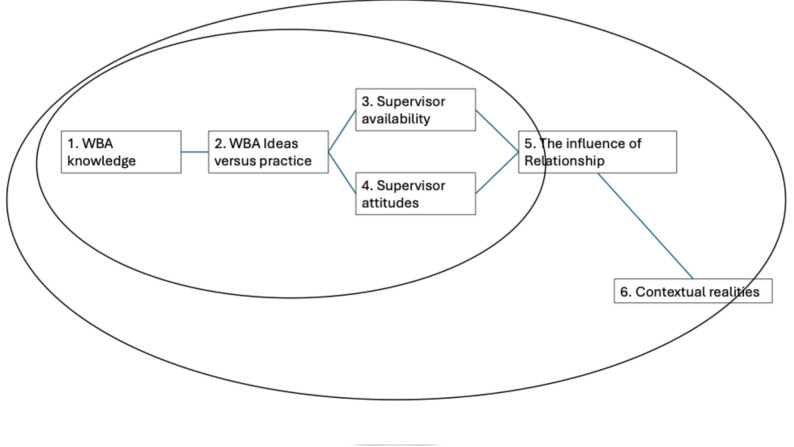
A diagrammatic representation of the emergent, interlinked themes

There were six distinct but cohesive themes that emerged from the analysis process. These are elaborated in the text and presented diagrammatically in Fig. 1. In summary, the key themes describe gaps in the knowledge of WBA, perceptions that expressed uncertainty about WBA as an idea versus a practice, perceptions of supervisor attitudes, practical issues of supervisor availability, the supervisor-trainee relationship, and the final theme acknowledging that the relationships and activities take place within a contextual reality.

#### Gaps in knowledge

Many trainees could accurately define WBA. However, during the first round, when they described what was happening in practice it was clear that they had certain misconceptions about what WBA was. For example, one participant provided the following scenario typical of other responses as exemplary of WBA in their respective discipline:

*‘We don’t necessarily have portfolios […] we have tutorials […] they give you a short scenario… you do your questioning;* *you can do your examination* *and then afterwards we discuss…’* (Participant 05 − 01).

During the second round some participants had shifted in their perceptions of what WBA is to a more accurate conception:

*‘[…] the current system is sort of logbook-based […] you know it doesn’t assess competency […] So at least* *with the work-based… you actually get to a point where you are* *confident and competent’* (Participant 01–13).

#### A good idea, but not sure about practice

In round 1 focus groups trainees were, in general, remarkably positive about WBA, perceiving it, as one participant put it, as ‘a real game changer’ and remarking on its potential to improve the frequency of feedback and thus provide trainees with an accurate sense of their strengths and weaknesses prior to the exit examination.

*‘It [WBA] is a game changer* *[…] It helps when we get feedback from our supervisors*, *it helps us see where we need to improve* *while we have time on an ongoing basis* *rather than to put everything at the end* *when we study for the final.’* (Participant 01–04).

Another trainee commented on their expectation that real-time assessment would provide a more accurate picture of their abilities than an exit examination:

*‘[…] I mean what you do throughout four years is* *more representative than one exam*, *so that’s how I understood it* (Participant 02–04).

One trainee emphasized their perception that on-going workplace-based assessment would help them identify strengths and weaknesses from moment to moment. Again, the desirability of the fact that this could happen before high stakes exit examinations was emphasized.

*‘[…] every little mistake you do*, *they [supervisors] can correct it right on the spot*, *not after an exam. So*, *you get to improve on your weaknesses as you go*, *unlike waiting until the end.’* (Participant 06 − 01).

In round 2 focus groups, participants were still willing to engage with WBA activities, but some reported that although some progress had been made it was too little and not adequately implemented:

*‘[…]* *they’re like where do I tick to say you are competent* *or are not competent*,* because* *they didn’t have those marking rubrics*.*’* (Participant 06 − 01).

When participants were pressed about the reasons for slow or inadequate implementation they cited the need for leadership from the top, but they were also quick to follow this up by mentioning constraints on time and human resources as limiting factors to successful implementation.

‘*[…] we do kind of expect [WBA] to be* *implemented top down*, *you know*,* from consultant level down to us…And then* *I’m not sure like how intensive*,* labour-wise*,* this work-based assessment is….’* (Participant 06–11).

At some institutions participants said that *no* progress had been made between the first introductory workshop to WBA approximately six months prior and our second focus group together:

*‘No*, *we haven’t actually started*; *I think we’ve only had one encounter with the specialist.’* (Participant 05–06).

#### Supervisor availability

Overall, participants described a situation in which, for a variety of reasons, they perceived supervisors as often not present to observe them in the workplace, leading to concern among trainees about how WBA would work in practice. In response to a question about general trainee anxieties about WBA, one participant said of their peers and their own experience:

*‘…often the consultants are just not there*.*’* (Participant 07–07).

This general point about a lack of supervisor availability was reinforced by other participants too.

In the second round of focus group interviews, supervisor availability remained an issue:

*‘Mine [participant’s experience of WBA] hasn’t really changed since our initial interview [four months prior]. So*, *struggling with getting time from consultants to do the actual assessments*, *mostly having to rely on like fellow registrars to help me out […]’* (Participant 02 − 01).

Participants from other disciplines and institutions echoed this sentiment.

However, there were examples of positive change getting WBA up and running. For example, one participant described the situation in their discipline in the following way:

*‘We have started with the program*,* we have a link that after each rotation*,* preferably on a weekly basis*, *we sit with our supervisors*, *and we assess each other*,* and* *we get feedback from them*.*’* (Participant 06 − 02).

#### Supervisor attitudes

Some data revealed a more serious perception/experience of supervisor unwillingness to engage in the teaching and learning process. One participant even saw WBA as a way to ensure supervisor engagement and accountability in teaching.

*‘[…] what* *we want [is] better teaching* *[…] and this [WBA] is one of the ways that I think we’re sort of* *force […] consultants to be more active* *in our training.’* (Participant 04 − 01).

Another trainee, however, with more existing experience of WBA in their discipline (WBA had been implemented a few years prior to the current study) felt that supervisors were not more willing to teach despite the presence of WBA requirements, with the trainee facing the consequences of inadequate activities in their portfolio:

*‘My feeling* *now that we’ve been doing it […]* *is actually negative*. *I feel that there’s* *no consultant willingness* *to participate […] You find that you get to the end of a rotation and you have anxiety because you know that for your academic record you need five […] and* *it’s not in your control to force anyone to do it*.*’* (Participant 04–04).

This comment speaks to a more general feeling of disempowerment among trainees vis-a-vis the scope of their personal agency and their ability to secure the teaching/assessment they need, if WBA is to become an entrenched reality of their training.

In round 2, participants described both ongoing unwillingness on the part of supervisors to do observations (as above): For example:

*‘The* *concern would be the consistent participation of the consultants* *at this stage. As I mentioned*,* everything has been planned*,* but* *nothing’s actually been set out to be done*.*’* (Participant 01–14).

This was not true for all contexts, where in some instances supervisor willingness had improved. For example, one participant reported:

*‘Our* *consultants have been implementing it* *[…] At first*,* they teach it and then they assist us doing it and then they evaluate basically how far they think you are supposed to be able to go with procedure*,* etc.’* (Participant 03–09).

#### Influence of the supervisor-trainee relationship

Most participants expressed concern in round 1 about the role that the supervisor-trainee relationship might play in assessment outcomes particularly in cases where that relationship was poor.

*‘You find that with* *the ones [consultants] that was [sic] nice to you*, *even at work*, *she or he volunteers to teach you* *[…]. As opposed to someone that is very much mean*,* is* *quite uninterested in teaching you[…]’* (Participant 07 − 04).

Some also expressed the reverse, that a good relationship might positively influence an assessment:

*‘[…]* *if you have a good relationship* *with your consultant or facilitator*,* you’re probably* *most likely to get good marks*, *good reviews. And if you have a* *bad relationship*, *then* *it would affect the mark* *that you would get.’* (Participant 05 − 02).

One participant described the potential for victimization, (mis)perceived to be inherent in WBA, explaining what they perceived to be the generally negative attitude to WBA among trainees they knew:

*‘I think* *victimisation is going to be a very big factor* *[…] The reason why they had a negative attitude towards it [WBA] was the thought of the fact that some of them already had* *bad relationships with their consultants*. *So*,* there was a* *fear* *now with the whole victimisation thing.’* (Participant 03–03).

Such experiences and the anxieties they evoked were expressed across institutions and disciplines, with supervisor opinions instigating stigma within teams:

In the six-month gap between the introductory WBA workshop and the first round of focus group interviews and the second round, not much had shifted:

*‘[…] we do have very small teams*,* so you’ll have for example* *two consultants in a department*, *and you may or may not be able to work with both of them […] or this is* *someone that I think has biases against women* *[…]’* (Participant 04–04).

A significant concern of trainees was the fact that exams can be standardized and controlled in a way that WBA cannot. Significant anxiety was expressed in this regard.

*‘I had a concern about the* *universality of exams*. *Because now if you’re going to take out the writing exams […] it* *needs to be standardized* *so that whatever that is being taught in Cape Town is taught in Durban*,* is taught in Umtata […]’* (Participant 07 − 4).

#### Contextual realities

Unsurprisingly in both round one and round two focus group participants expressed concerns about how variable clinical settings would impact learning and assessment:

*‘[…] we are* *not previously disadvantaged; we are still disadvantaged*. *That’s a fact in terms of the […] equipment and things that we need. So*,* there are a* *lot of things that we do not have*. *That would be my concern for this work-based assessment […]’* (Participant 07 − 01).

## Discussion

Our findings emphasize the importance of knowledge of WBA, the need for a structured workplace and engaged faculty, the key role that the supervisor-trainee relationship plays, and overwhelming contextual factors that may influence teaching-learning opportunities. Some of these findings are particularly relevant to Lower- and Middle-Income Countries (LMICs) like South Africa, while other are broadly relevant across local and international contexts. This discussion section is divided into two parts: the first discusses findings of broad relevance, the second findings specific to LMIC contexts such as South Africa.

### a. Findings of general relevance

Among the trainees we interviewed for this study there was still a lack of conceptual clarity about WBA. For example, some perceived WBA as summative rather than formative. A previous study in the SA context showed that misconceptions about WBA exist among supervisors too [3]. Having a clear understanding of WBA is likely to be critical in terms of motivating trainees to adopt it and make the best use of it. The lack of clarity among some members of this cohort suggests that some form of training specifically for trainees as to the purpose and workings of WBAs will be a necessary ingredient in its success going forward. We already recognize that this is the case for supervisors [3]. Confusion about the difference between formative and summative assessments in the context of WBA is common across international medical education settings, not just in the South African case. Research on workplace-based assessments internationally has found that even when WBAs are intended to be formative, learners often perceive them as summative or high-stakes, which can undermine their use for learning and feedback [31]. 

In addition to a lack of conceptual clarity about WBA there was also a lack of clarity around what the trainee and supervisor roles are in the learning exchange of WBA. Trainees in this study described themselves in terms that suggested they lack agency or a sense of responsibility in terms of securing access to the activities they need to engage with to learn and progress. According to trainees in this study, supervisors also seem not to be clear about their roles and responsibilities in doing observations and offering feedback. Although there is no literature that looks at this topic explicitly in the context of implementing WBA, best practice guidelines on supervision and learning do strongly suggest that clarity of roles and concepts in postgraduate medical education is essential for fostering effective learning [32]. 

Effective WBA in postgraduate medical education is fundamentally dependent on the quality of the relationships between supervisors and trainees. Ideally, this relationship is characterized by trust and open communication; it is crucial for facilitating meaningful feedback and promoting learning. The evidence suggests that trainees are more receptive to feedback and more likely to engage in reflective practice when they trust their supervisors and perceive feedback to be constructive and supportive [17]. In the international context, Castanelli and colleagues [13] highlight that even though the hierarchical nature of the supervisor-trainee relationship can pose challenges, when approached intentionally and proactively it provides trainees with opportunities for growth and learning. Their study emphasizes the importance of supervisors using their authority to create a safe learning environment where trainees feel comfortable seeking feedback and discussing their performance. This holds lessons for WBA implementation in SA, with faculty development as a key component of that process.

One critical area in which relationships can be improved is through the capacitation of supervisors to give effective feedback. Feedback, like other forms of communication, is a skill that can be learned [33, 34]. Supervisors who are trained in feedback delivery techniques are better equipped to offer feedback that is timely, specific, and actionable, thereby enhancing the learning experience for trainees and improving supervisor-trainee relationships [35]. 

### b. Findings relevant to South Africa and other LMICs

In contexts like South Africa, and the global South more broadly, contextual issues like lack of resources, including equipment, time, and personnel, are an ‘on the ground’ reality where the training of postgraduate medical specialists takes place. Strong organizational frameworks and structured implementation are pivotal for the success of WBA in postgraduate medical education. In South Africa, supervisors have identified structural readiness, staff capacity, and quality assurance as critical factors influencing WBA implementation [3]. Similarly, a study from Bhutan reported that while WBA was well-received, challenges such as time constraints and limited faculty capacity impeded its effective execution [36]. These findings point to the centrality of strong organizational support and structured implementation, implying that early engagement with senior management in academic institutions and health facilities is a key success factor. As the implementation unfolds, this collaboration between educational innovation and management structures could offer interesting research opportunities in low-resource settings.

Critical contextual awareness is necessary in the SA and global southern context when implementing WBA. A successful program for the implementation of WBA cannot only be based on the realities of well-resourced universities; it must also take into consideration the realities of less well-resourced institutions. This raises a broader question about equitable access to training resources, especially when measured against the expected equality of training outcomes [21]. Discussions and decisions about curricula and policy will have to take these inequalities into consideration and make sure that decisions are context-sensitive, and inclusive of all facilities offering postgraduate medical specialist training in South Africa.

### Strengths and limitations

An abiding strength of this study is its wide national representation across all specialist training facilities in SA. This ensured that the dataset generated was reflective of widespread opinions and perspectives. The rigor applied to the data collection and analysis process provided findings that are trustworthy.

The limitations are related to the qualitative nature of the study design, in that the sample of participants was not constituted in a manner that would provide findings that are generalizable to the broader SA population of trainees. A second limitation is reflected in the attrition of participants between the two phases of the data generation process.

Attrition is a contextual limitation inherent to longitudinal qualitative research. In this study fewer trainees participated in the second round of focus group discussions, which may have reduced the breadth of perspectives captured at that later time point. In line with qualitative methodological principles, we interpret attrition reflexively rather than statistically, recognizing that ongoing clinical workload, service pressures, and the realities of postgraduate training in a resource-constrained health system likely influenced participants’ continued availability. These same contextual factors are analytically relevant to the study’s findings, particularly regarding supervisor availability, workload, and implementation feasibility, and therefore do not undermine the trustworthiness or interpretive validity of the results.

## Conclusion

This qualitative study explored the perspectives of medical specialist trainees of early implementation of WBA in the SA context. Six key themes emerged from the data, indicating areas of opportunity and risk in ongoing implementation efforts. The findings hold value for leaders in health professions education and healthcare systems that host medical specialist training programmes and suggest the following concrete and actionable implications for implementation:

- Establish role-clarity (i.e. train supervisors and trainees in the specifics of their roles in WBA).
- Ongoing faculty development to enhance the quality of feedback.
- Protected time for observation.
- and context-sensitive quality assurance.

Currently, all supervisors performing WBA are offered standardized training within their institution. The training has been validated by a group of experts and piloted across different disciplines and different contexts on the training platform. The quality of supervisor assessments is monitored through the trainees’ portfolio, which allows for the Head of Department to have oversight of all portfolios, including the feedback provided to students by supervisors.

- We propose that this quality assessment process forms part of the annual performance appraisal of all supervisors.

Further research should focus on ways to enhance supervisor-trainee relationships, the influence of training platform inequities on training outcomes, and ongoing faculty development to enhance the implementation and sustainability of WBA in low-resource contexts. As such, expansion of the project into other African countries needs to be explored.

## Supplementary Information

Supplementary Material 1.

Supplementary Material 2.

Supplementary Material 3.

Supplementary Material 4.

## Data Availability

The datasets used and/or analysed during the current study are available from the corresponding author on reasonable request.

## Declarations

### Clinical trial number

No clinical trial number.
