# Supplementary material for: The introduction of workplace-based assessment into postgraduate medical training in South Africa: trainee perspectives
Source: BMC Med Educ. 2026 Feb 21;26:515. doi: 10.1186/s12909-026-08792-w (PMC13032551; doi:10.1186/s12909-026-08792-w)
Supplement: Supplementary file 1 — Supplementary Material 1. [file 12909_2026_8792_MOESM1_ESM.pptx]

## Slide 1
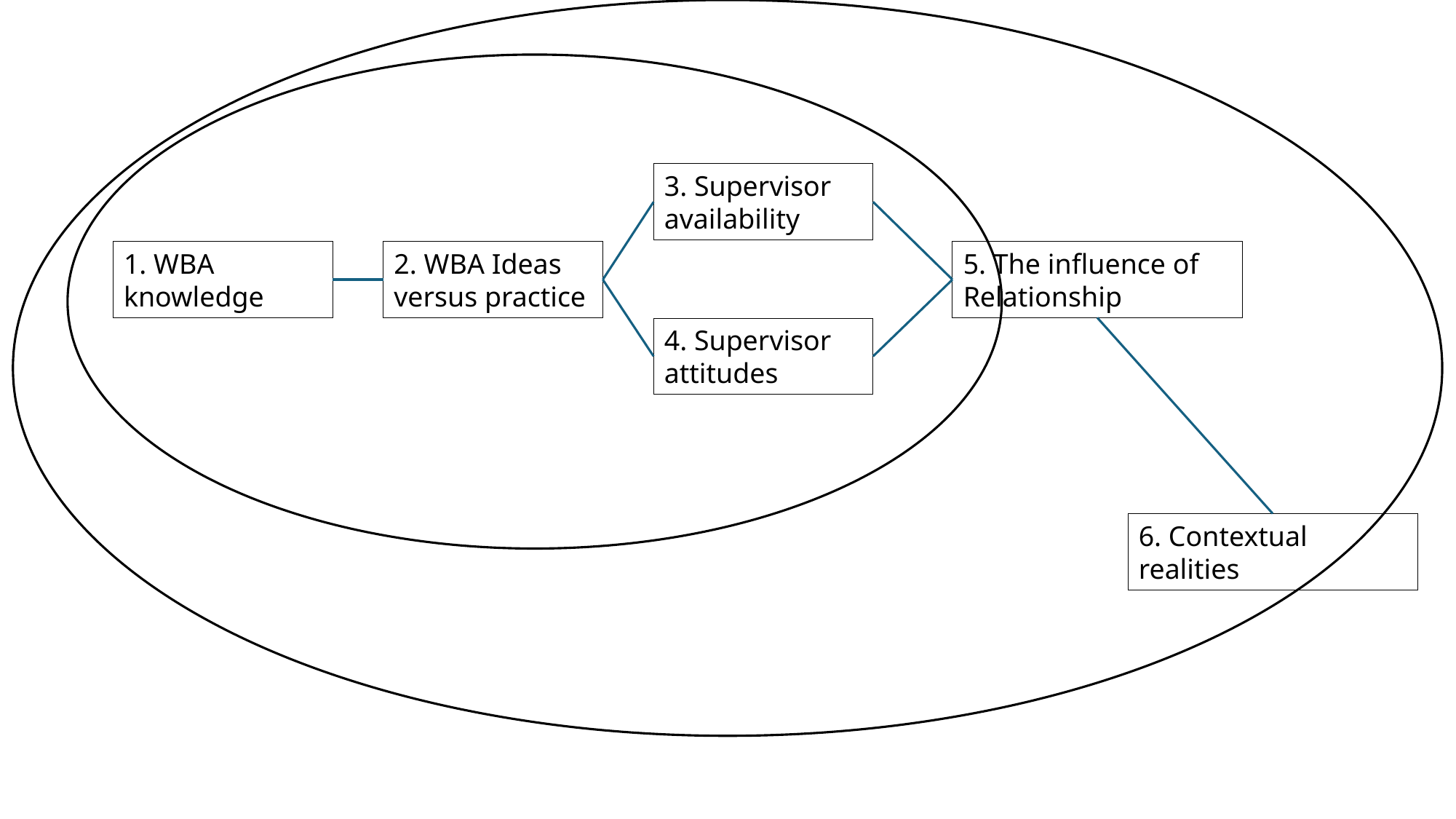

3. Supervisor availability
5. The influence of Relationship
1. WBA knowledge
2. WBA Ideas versus practice
4. Supervisor attitudes
6. Contextual realities
