# Supplementary material for: The introduction of workplace-based assessment into postgraduate medical training in South Africa: trainee perspectives
Source: BMC Med Educ. 2026 Feb 21;26:515. doi: 10.1186/s12909-026-08792-w (PMC13032551; doi:10.1186/s12909-026-08792-w)
Supplement: Supplementary file 2 — Supplementary Material 2. [file 12909_2026_8792_MOESM2_ESM.docx]

**Workplace-based assessment (WBA) registrar focus group questions:**

- What is your understanding of what WBA is? Describe.
- What are your expectations/concerns/fears/concerns about WBA post the onboarding workshop?
  - What do you expect WBA to be like for you as a registrar?
  - How do you expect it will benefit you? What do you expect to get out of it?
  - What are your fears and concerns about WBA as a registrar?
- Reflect on your experiences…positive and negative
  - Describe your experience of WBA (in the workplace and/or at this workshop)?
  - Describe any negative aspects of WBA
  - Describe any positive aspects of WBA
- What did you learn? What impact did this have?
  - What did you learn through the workshop about WBA?
  - How is this the same or different from your prior perceptions or experiences of WBA? Elaborate
  - How are you feeling about WBA now? Fears and concerns?
- What has changed? What would you like to change?
  - What is your perception of WBA post the workshop?
  - What do you think still needs to change for WBA to be beneficial for you and other registrars?
  - What needs to change for WBA to work?
