# Supplementary material for: The introduction of workplace-based assessment into postgraduate medical training in South Africa: trainee perspectives
Source: BMC Med Educ. 2026 Feb 21;26:515. doi: 10.1186/s12909-026-08792-w (PMC13032551; doi:10.1186/s12909-026-08792-w)
Supplement: Supplementary file 4 — Supplementary Material 4. [file 12909_2026_8792_MOESM4_ESM.docx]

**COREQ Check List**

| **COREQ Domain** | **Item** | **Description (as reported in manuscript)** |
| --- | --- | --- |
| **Research team & reflexivity** | Interviewer | Focus group discussions facilitated by ED, the lead qualitative researcher. |
|  | Credentials | ED is a sociologist with formal training and extensive experience in qualitative research. |
|  | Occupation | Academic researcher, University of Cape Town. |
|  | Gender | Female. |
|  | Experience | Experienced in qualitative health professions education research. |
|  | Prior relationship | No prior relationship between interviewer and participants. |
|  | Participant knowledge | Participants were informed that the interviewer was not a clinician, supervisor, or assessor and had no role in training decisions. |
|  | Reflexivity | Interviewer’s position outside the medical hierarchy was explicitly considered to minimise power differentials. |
| **Study design** | Methodological orientation | Inductive qualitative design using reflexive thematic analysis |
| **Participant selection** | Sampling | Purposive sampling of postgraduate trainees from institutions participating in the national WBA pilot. |
|  | Recruitment | Invitation following attendance at institutional WBA onboarding workshops. |
|  | Sample size | Phase 1: 84 participants (11 FGDs); Phase 2: 45 participants (7 FGDs). |
|  | Non-participation | Attrition between phases acknowledged and discussed as a limitation inherent to longitudinal qualitative research. |
| **Setting** | Data collection setting | Online focus group discussions conducted via Zoom. |
|  | Non-participants | No non-participants present. |
|  | Sample description | Postgraduate specialist trainees across 29 specialties at 7 South African universities. |
| **Data collection** | Interview guide | Semi-structured guide developed by the research team and piloted prior to use. |
|  | Repeat data collection | Longitudinal design with repeat FGDs approximately one year apart where feasible. |
|  | Recording | Audio-recorded with participant consent. |
|  | Field notes | Field notes taken to document contextual observations. |
|  | Duration | Approximately 60 minutes per focus group. |
|  | Saturation | Thematic saturation reached early and confirmed through ongoing analysis. |
|  | Transcript return | Transcripts not returned to participants. |
| **Analysis** | Number of coders | Initial independent coding by ED and TR; full dataset coded by ED. |
|  | Coding process | Codes grouped into categories and refined into themes through iterative team discussion. |
|  | Theme derivation | Themes derived inductively from the data. |
|  | Software | Manual qualitative analysis; no software used. |
|  | Member checking | Not undertaken; credibility addressed through reflexivity, team analysis, and rich data excerpts. |
| **Reporting** | Quotations | Verbatim quotations presented with participant identifiers. |
|  | Data–finding alignment | Clear alignment between data excerpts and analytic claims. |
|  | Themes | Major themes and sub-themes clearly presented and supported by data. |
